# A Huge Calcified Supratentorial Ependymoma: A Case Report

**DOI:** 10.7759/cureus.37493

**Published:** 2023-04-12

**Authors:** Kivanc Yangi, Ahmed Yasin Yavuz, Gokhan Percinoglu, Buse Aki, Suat Erol Celik

**Affiliations:** 1 Neurological Surgery, Prof. Dr. Cemil Tascioglu City Hospital, Istanbul, TUR; 2 Pathology, Prof. Dr. Cemil Tascioglu City Hospital, Istanbul, TUR

**Keywords:** pineal region, supra-tentorial tumor, pineal region tumor, supratentorial ependymoma, tanycytic ependymoma

## Abstract

Tanycytic ependymoma has been marked as Grade II by the World Health Organization (WHO), requiring considerable treatment. However, according to the fifth edition of the WHO Classification of Tumors of the Central Nervous System published in 2021, tanycytic ependymoma is no longer identified as a subtype of ependymoma. Herein, we offer an unusual case of a supratentorial ependymoma, previously tanycytic ependymoma. Which radiologically mimic pineal region tumors; however, they pathologically mimic meningiomas, schwannomas, medulloblastomas, or astroblastomas.

A three-year-old girl presented to our neurosurgery department with sudden onset gait disturbance and balance impairment; we detected no additional neurologic deficit. Magnetic Resonance Imaging (MRI) revealed a giant, multilobulated, well-circumscribed right pineal mass, approximately 4.5 x 4.5 x 4.5 cm in size, crossing the midline and extending posteriorly, invading the pineal region. The initial diagnosis was a pineal region tumor. Following gross-total resection of the tumor, pathology reports showed tanycytic ependymoma. Postoperatively the patient's gait disturbance was improved, and there was no balance impairment. Follow-ups at three and six months, no sign of recurrence has been encountered.

Our case demonstrates that supratentorial ependymomas may also occur in the pineal region and requires an accurate neuropathologic diagnosis. Early accurate diagnosis is essential; since those tumors may be related to a wide range of prognoses and necessitate different treatment modalities.

## Introduction

Ependymomas are one of the most common malignant intracranial neoplasms in children. Although they can arise in practically every area of the neuroaxis, the posterior fossa is the most frequent location, followed by the supratentorial region and the spinal cord [1]. We discuss tanycytic ependymoma (TE) because this case occurred before the new guideline announcement, and the first pathology report resulted in tanycytic ependymoma. TE (World Health Organization (WHO) grade II) is an uncommon variant of ependymoma. There is limited information describing the tumor and its management in the literature; therefore, no particular guidelines are published. We report a three-year-old female patient with a vast calcified supratentorial ependymoma, previously called tanycytic ependymoma, located in the pineal region.

## Case presentation

**Figure 1:**
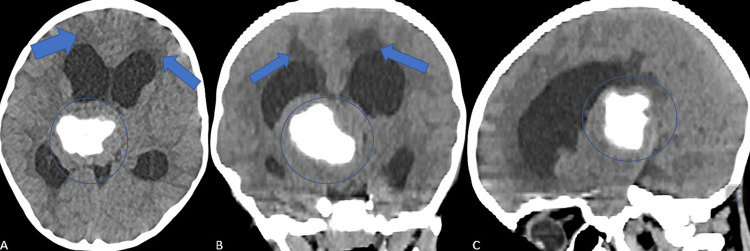
Preoperative non-contrast-enhanced CT scan of a supratentorial ependymoma. A: Axial scan showing that triventricular hydrocephalus caused by the mass lesion was observed. Hypodensities secondary to transependymal CSF transmission were observed in bilateral periventricular white matter (blue arrows). Intralesional hyperdensities were also observed (blue circle). B: Coronal scan showing that from the superior part of the third ventricle, a mass lesion extending to the fourth ventricle, particularly the right side. The interventricular septum cannot be distinguished, the total size is approximately 4.5 cm, and its solid components are isodense with the cortex. The lesion also showed central calcified portions (blue circle). Transependymal CSF transmission was observed adjacent to the superior borders of the lateral ventricles (blue arrows). C: Sagittal scan showing a centrally calcified mass lesion (blue circle), compressing the lateral ventricles. The differential diagnosis should consider ependymoma, central neurocytoma, and pineal region tumors.

A three-year-old girl with no history of congenital defects or comorbidities presented to neurosurgery with gait abnormalities and rapid balance impairment, which had developed over four months. Following a physical examination, we detected no further neurological impairment. Non-contrast-enhanced computerized tomography (CT) showed a midline isodense intracranial lesion, including hyperdensities near its center, approximately 4.5 × 4.5 × 4.5 cm in diameter, in the pineal region next to the quadrigeminal cistern (Figure 1)

**Figure 2:**
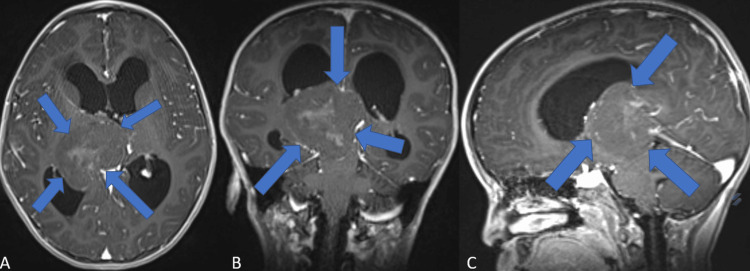
Preoperative contrast-enhanced cranial MRI of a supratentorial ependymoma. A: Axial T1-weighted image with contrast material enhancement showing an isointense pineal lesion displaying intralesional calcified hemorrhagic hyperintense signals, compressing both the lateral and third ventricles. Blue arrows indicate the borders of the lesion. B: Coronal T1-weighted image with contrast material enhancement showing the isointense pineal lesion displaying intralesional calcified/hemorrhagic hyperintense signals, compressing the adjacent structures. Blue arrows are describing the borders of the lesion. C: Sagittal T1-weighted image showing an isointense pineal mass displaying intralesional hyperintensities, compressing the surrounding structures and resulting hydrocephalus. Blue arrows indicate the borders of the lesion.

Subsequent contrast-enhanced magnetic resonance imaging (MRI) revealed a calcified hemorrhagic, well-circumscribed lesion in the right pineal region and isointense on T1-weighted sequences (Figure 2). Both lateral and third ventricles showed an increased size, and tetraventricular hydrocephalus was observed. The neuroradiologic differential diagnosis included midline tumors such as pineal region tumors. A spinal contrast-enhanced MRI was also performed and showed no sign of pathologic enhancement. Surgical exploration was planned.

**Figure 3:**
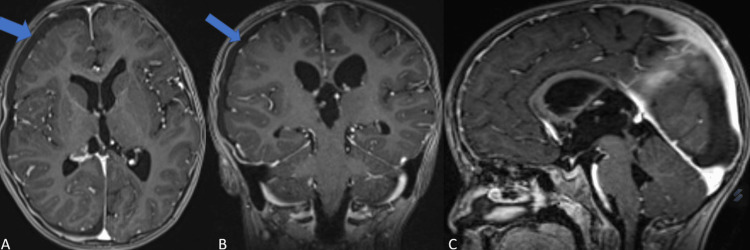
Postoperative contrast-enhanced MRI of a supratentorial ependymoma. A: Axial scan showing the complete removal of the tumor. All the ventricles appear relieved. As a secondary effect of surgery, the right frontoparietal subarachnoid space is enlarged (blue arrows). The enlargement can be defined as postoperative subdural hygroma (blue arrows). B: Coronal scan showing the gross-total resection. No residual tumor sign was observed. The right frontoparietal subarachnoid space was enlarged (blue arrow). C: Sagittal scan showing a marked regression in accompanying triventricular hydrocephalus and total regression in transependymal periventricular edema.

After a right parieto-occipital craniotomy, the tumor was grossly totally resected. Macroscopically, the tumor was a well-circumscribed, slightly firm configuration, a gray-colored mass that crossed the midline and extended posteriorly, invading the pineal region. Although it was highly adherent to the surrounding vascular structures and parenchymal tissues, gross-total resection was accomplished (Figure 3). The vascular structures have been preserved, and no obvious bleeding was noticed from the main vessels during the operation.

**Figure 4:**
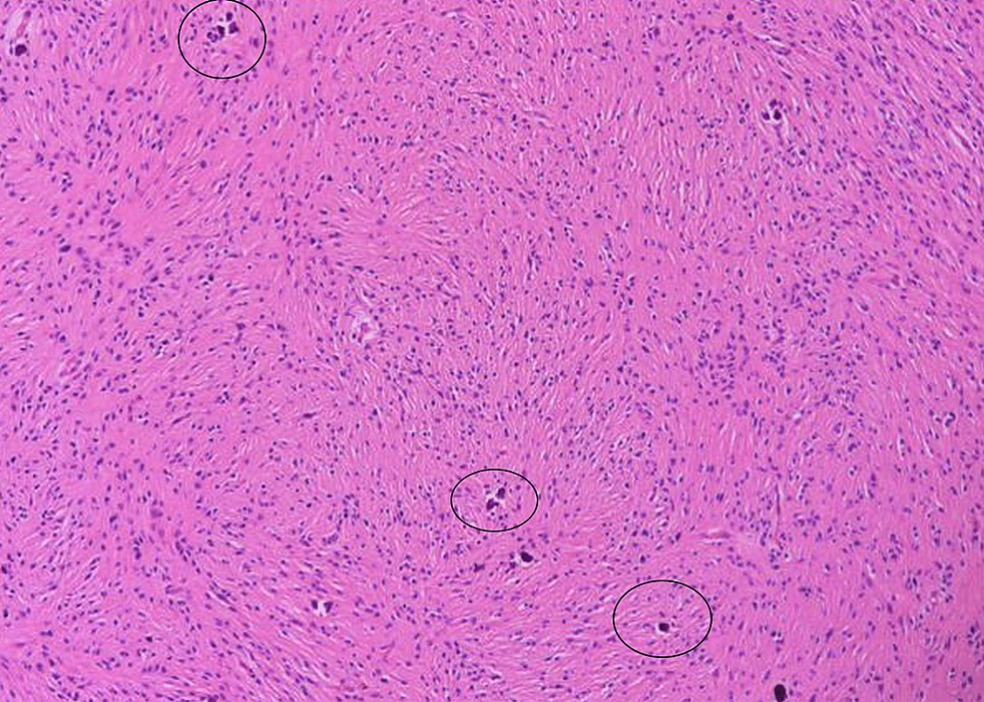
Pathologic specimen image 1 Neoplastic spindle cells with oval to elongated nuclei arranged in interlacing fascicles (black circles) H&E, x100.

**Figure 5:**
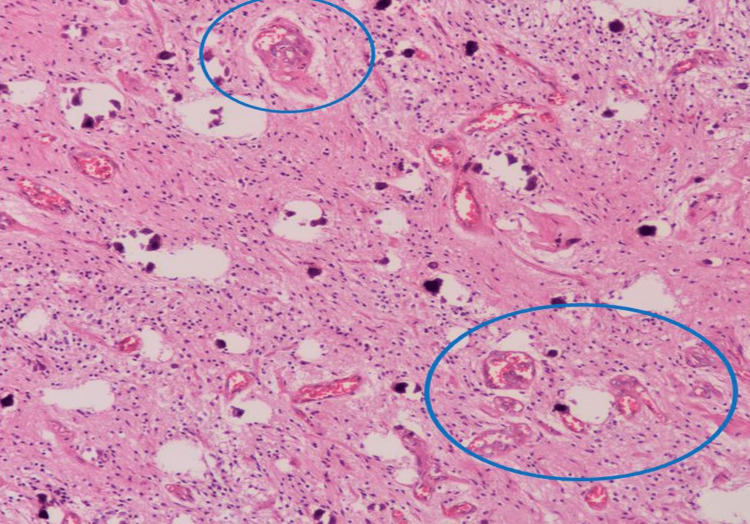
Pathologic specimen image 2 High-power magnification of vascular endothelial proliferation shows the prominent layering of endothelial cells. This phenomenon is often dissociated from lumen formation (blue circles), H&E, x100.

**Figure 6:**
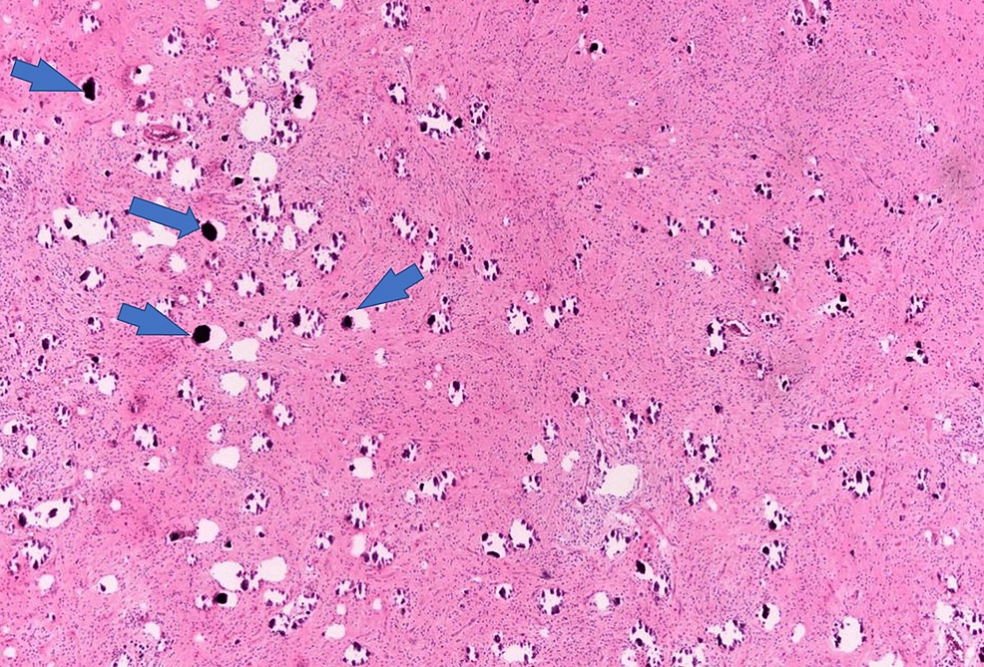
Pathologic specimen image 3 Numbers of psammoma bodies between tumor cells (blue arrows), H&E, x40

Pathologic examination revealed a 44 cc volume of cream-yellow curettage material from the mass spreading from the midline to the pineal region at the third ventricle level. Light microscopy showed that the tumor consists of spindle cells with oval and elongated nuclei with speckled chromatin and small nucleoli arranged in interlacing fascicles (Figure 4, black circles). The tumor was moderately cellular. Among the tumor cells, vascular endothelial proliferation areas were noted, showing prominent layers of endothelial cells (Figure 5, blue circles). In almost all sections, the number of psammoma bodies was seen (Figure 6, blue arrows).

**Figure 7:**
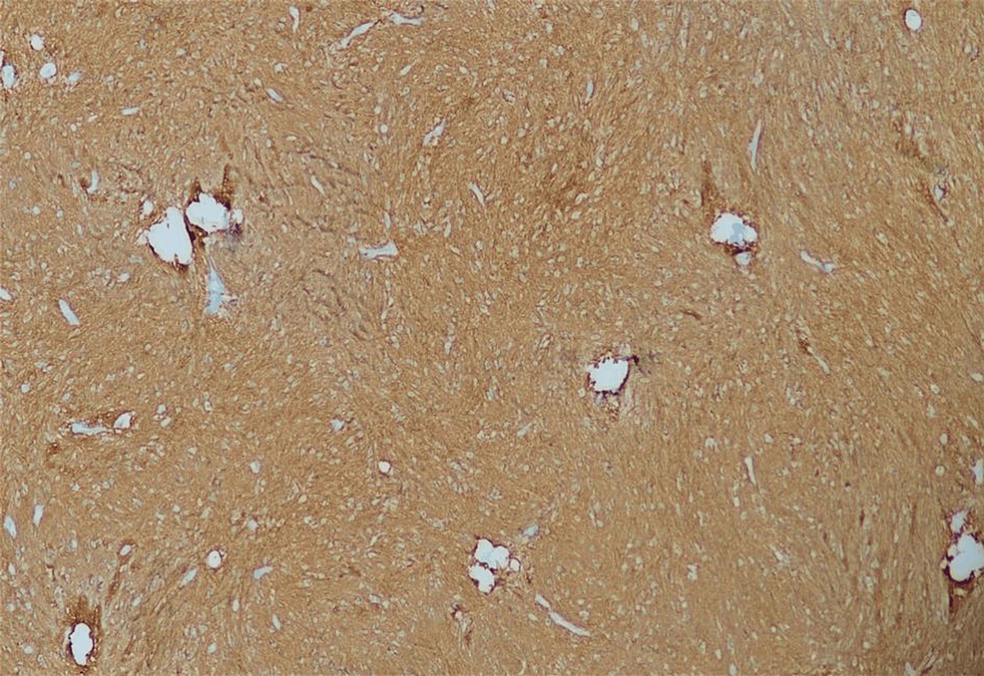
Pathologic specimen picture (GFAP) Neoplastic cells diffuse strong immune reactions for glial fibrillary acidic protein (GFAP), x100.

**Figure 8:**
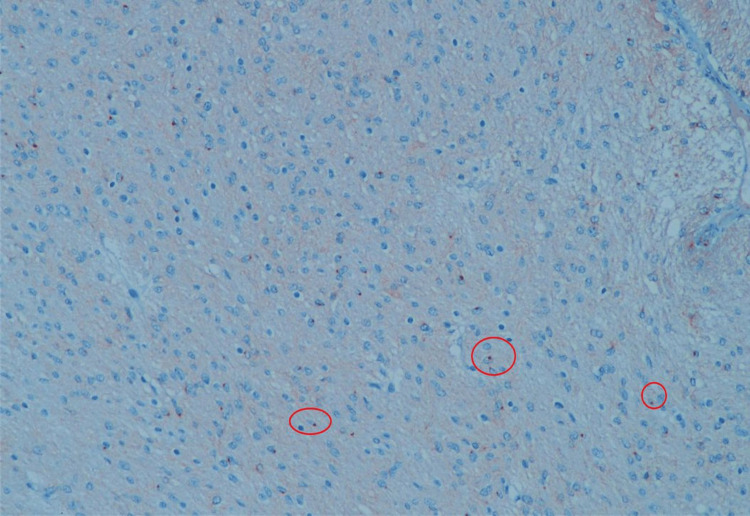
Pathologic specimen picture (EMA) Tumor cells show characteristic perinuclear dot-like positivity within the cytoplasm with epithelial membrane antigen (EMA, red circles), x200.

Mitotic activity was not elevated, and Kİ67 proliferative index was 2-3%. Palisading necrosis and hypercellular areas were not observed. Tumor cells were diffusely immunoreactive for glial fibrillary acidic protein (GFAP) and showed characteristic perinuclear dot-like positivity within the cytoplasm with epithelial membrane antigen (EMA) (Figure 7) and (Figure 8, red circles). Pathology reports have resulted in tanycytic ependymoma. However, tanycytic ependymoma is no longer identified as a subtype of ependymoma, according to the fifth edition of the WHO Classification of Tumors of the Central Nervous System published in 2021 [2]. It has been re-classified as a supratentorial ependymoma according to its anatomical localization. However, based on the microscopical findings, meningiomas, schwannomas, medulloblastomas, and astroblastomas should be considered in pathologic differential diagnosis.

Left hemiparesis was occurred after the operation. The muscle strength of the left upper and lower extremities was graded as a 2/5 on a muscle strength scale. However, the patient's gait disturbance improved, and there was no balance impairment. The patient consulted to physiotherapy department and started muscle-strengthening exercises. On the first-month follow-up of the patient, muscle strengths were found to be 4/5 on the left side. Postoperative MRI scans showed no residual tumor. No sign of recurrence has been encountered at the patient's postoperative third and sixth-month follow-ups. However, it should be noted that ependymoma frequently returns within two to three years of resection because of insufficient surgery. For this reason, the patient will be monitored with MRI scans at three to six-month intervals for the first three postoperative years, then annually.

## Discussion

Ependymomas are heterogeneous tumors belonging to gliomas [3]. Ependymoma can arise from any part of the central nervous system. They account for 5-6% of all intracranial gliomas, and 69% occur in children. Tanycytic ependymomas are rare but benign. There are few cases of ependymoma variants presented in the literature. The treatment of preference for tanycytic ependymomas is gross-total resection. However, postoperative radiation therapy should be considered when gross-total resection cannot be accomplished. Since these tumors are considered benign, chemotherapy is usually not accepted as a treatment modality.

Tanycytic is no longer recognized by name as a subtype of ependymomas. According to the 2021 WHO Classification of Central Nervous System Tumors, ependymal tumors are classified into five groups: supratentorial ependymoma, posterior fossa ependymoma, spinal ependymoma, myxopapillary ependymoma, and subependymoma [2]. Therefore the pathology of this tumor was re-examined in this recent case, and the report resulted in supratentorial ependymoma based on the microscopical findings and anatomical localization. Molecular analysis was unavailable in our center. According to the 2021 WHO Classification of Central Nervous System Tumors, ependymal tumors can be classified according to the histopathologic features and anatomical locations when molecular analysis is unavailable.

Of juvenile ependymomas, 90% are located intracranially, with 70% being infratentorial and 30% supratentorial. Supratentorial ependymomas are grade II to grade III tumors [2] typically present inside the brain parenchyma, possibly due to the ependymal stem cell trapping during the embryologic phase. These tumors are frequently well- circumscribed because of the entrapment. In other words, they have discrete borders with adjacent structures [4].

On the other hand, malignant tumors, in particular, may present acutely due to intratumoral bleeding. Intratumoral calcifications were found in this case, although there was no evidence of bleeding. Supratentorial ependymomas may be linked to the ventricular system and extend into the cerebral cortex. However, neither the ventricular system nor the cerebral cortex was implicated in our case.

It is necessary to distinguish these tumors from pineal region tumors. Pineal region tumors of the central nervous system are rarely seen in the pediatric population. The most common subtypes are pinealoblastoma and germ cell tumors. Pineal region tumors have no pathognomonic CT or MRI imaging pattern. Contrast enhancement also varies; malignant neoplasms usually show more evident enhancement than benign ones [5]. This information demonstrates the challenging nature of distinguishing between pineal region tumors and supratentorial ependymomas originating in the pineal region with radiologic imaging results. In almost all cases, the primary treatment modality for pineal region tumors was surgery or biopsy, followed by adjuvant radiotherapy. According to some studies, the extent of surgical excision had no impact on overall survival [6]. A stereotactic needle and endoscopic third ventricular biopsy may be considered when the differential diagnosis includes pineal region tumors. In some cases, gross-total resection must be followed by adjuvant radiotherapy; however, in other cases, consistent adjuvant radiotherapy can be performed after the biopsy. Regarding the treatment modality of ependymomas, gross-total resection is the treatment of choice. If gross-total resection is achieved, a postoperative observation period is considered. In cases with subtotal resection, postoperative radiotherapy should be considered. Supratentorial ependymomas must be distinguished from pineal region tumors for accurate treatment planning.

Only a few cases of ependymomas in the pineal region are reported up to date. In a case from China, an ependymoma located in the pineal region (WHO grade II, according to the 2016 classification) was gross-totally resected, and the patient received no postoperative radiochemotherapy [7].

On the other hand, a study group from India presented a rare case of anaplastic ependymoma (WHO grade III, according to the 2016 classification) of the pineal region in a 42-year-old female patient. First, they performed ventriculoperitoneal shunt placement, then excised the tumor. They achieved a gross-total resection, and the patient underwent postoperative radiotherapy [8]. Furthermore, another group presented a 23-year-old male patient with low-grade ependymoma (according to their histopathological results, but WHO grading was not mentioned in the study) in the pineal region. They performed an endoscopic third ventriculostomy and biopsy and placed a left frontal external ventricular drain. They performed a second surgery for endoscopic tumor removal [9]. All these cases reported in the literature are before the new guideline announcement. According to our knowledge and based on the literature review, our case is the first reported case of a supratentorial ependymoma located in the pineal region after the new guideline announcement. We consulted the pathology department for a second examination of the tumor for a re-classification, and we updated our report.

## Conclusions

An unusual case of substantial calcified supratentorial ependymoma was presented in the pineal region of a three-year-old child. The ependymoma radiologically mimicked other pineal region tumors. Gross-total resection should be the treatment of choice. This case underlines the significance of early and accurate diagnosis and surgery to achieve an effective outcome and plan appropriate follow-up treatment. Further studies are required to hypothesize their radiological resemblance to midline tumors. When a sizeable centrally calcified midline lesion is observed on imaging, a surgeon should carefully evaluate the case, and ependymomas should also be considered in the differential diagnosis.

## Appendices

Limitations: Intraoperative pictures could not be obtained.
